# Dynamics of 5-carboxylcytosine during hepatic differentiation: Potential general role for active demethylation by DNA repair in lineage specification

**DOI:** 10.1080/15592294.2017.1292189

**Published:** 2017-03-07

**Authors:** Lara C. Lewis, Peggy Cho Kiu Lo, Jeremy M. Foster, Nan Dai, Ivan R. Corrêa, Paulina M. Durczak, Gary Duncan, Ashley Ramsawhook, Guruprasad Padur Aithal, Chris Denning, Nicholas R. F. Hannan, Alexey Ruzov

**Affiliations:** a Division of Cancer and Stem Cells, School of Medicine, Centre for Biomolecular Sciences, University of Nottingham, Nottingham, UK; b National Institute for Health Research (NIHR) Nottingham Digestive Diseases Biomedical Research Unit, Nottingham University Hospitals NHS Trust and University of Nottingham, Nottingham, UK; c New England Biolabs, Inc., Ipswich, USA

**Keywords:** 5-carboxylcytosine, definitive endoderm specification, DNA methylation, hepatic differentiation, hepatocytes, human pluripotent stem cells, 5-hydroxymethylcytosine, immunohistochemistry, TET1/2/3 proteins

## Abstract

Patterns of DNA methylation (5-methylcytosine, 5mC) are rearranged during differentiation contributing to the regulation of cell type-specific gene expression. TET proteins oxidize 5mC to 5-hydroxymethylcytosine (5hmC), 5-formylcytosine (5fC), and 5-carboxylcytosine (5caC). Both 5fC and 5caC can be recognized and excised from DNA by thymine-DNA glycosylase (TDG) followed by the subsequent incorporation of unmodified cytosine into the abasic site via the base excision repair (BER) pathway. We previously demonstrated that 5caC accumulates during lineage specification of neural stem cells (NSCs) suggesting that such active demethylation pathway is operational in this system; however, it is still unknown if TDG/BER-dependent demethylation is used during other types of cellular differentiation. Here we analyze dynamics of the global levels of 5hmC and 5caC during differentiation of human pluripotent stem cells toward hepatic endoderm. We show that, similar to differentiating NSCs, 5caC transiently accumulates during hepatic differentiation. The levels of 5caC increase during specification of foregut, peak at the stage of hepatic endoderm commitment, and drop in differentiating cells concurrently with the onset of expression of α fetoprotein, a marker of committed hepatic progenitors. Moreover, we show that 5caC accumulates at promoter regions of several genes expressed during hepatic specification at differentiation stages corresponding to the beginning of their expression. Our data indicate that transient 5caC accumulation is a common feature of 2 different types (neural/glial and endoderm/hepatic) of cellular differentiation. This suggests that oxidation of 5mC may represent a general mechanism of rearrangement of 5mC profiles during lineage specification of somatic cells in mammals.

## Introduction

DNA methylation (5-methylcytosine, 5mC) is an epigenetic modification associated with transcriptional repression contributing to the regulation of gene expression in a wide range of biologic settings.^1,2^ The patterns of DNA methylation are dynamic during development and cellular differentiation with 5mC being erased from and introduced to different sets of genomic regions specific for particular developmental stages.^1,2^ Thus, cellular differentiation is governed by both *de novo* methylation and demethylation of certain elements of the mammalian genome.^1,2,3^ Although the enzymatic machinery, which allows establishment and maintenance of the 5mC patterns, is relatively well characterized,^2,4,5^ the mechanisms of DNA demethylation were generally unknown until the discovery that Ten-eleven translocation proteins (Tet1/2/3) can oxidize 5mC to 5-hydroxymethylcytosine (5hmC), 5-formylcytosine (5fC), and 5-carboxylcytosine (5caC).^6,7,8^ These oxidized forms of 5mC (referred together as oxi-mCs) have been proposed to mediate dynamic changes of DNA methylation profiles during development via their potential involvement in both active and replication-dependent passive demethylation pathways.^9,10^ Importantly, both 5fC and 5caC can be recognized and excised from DNA by thymine-DNA glycosylase (TDG) followed by integration of non-modified cytosine into the generated abasic site by the components of base-excision repair (BER) pathway.^7,11^ Despite numerous indications that both TDG and oxi-mCs are important for development and cellular differentiation, the extent to which the TDG/BER-dependent demethylation is used in different developmental processes is still rather unclear.^12,13^ Thus, although this mechanism of active demethylation is operational in mouse embryonic stem cells (mESCs)^10,14^ and during mesenchymal-to-epithelial transition in somatic cell reprogramming,^15^ TDG-independent demethylation pathways seem to be involved in epigenetic reprogramming taking place during development of primordial germ cells (PGCs),^16,17^ and in mouse pre-implantation embryos.^18,19^

In our previous study, we demonstrated that 5caC accumulates during lineage specification of neural stem cells (NSCs) both *in vivo* and in cell differentiation experiments.^20^ Moreover, according to our data, TDG knockdown led to an increase in 5fC/5caC in differentiating NSCs, suggesting that the TDG/BER-dependent DNA demethylation pathway likely contributes to reorganization of the 5mC profiles occurring in this system.^20^ However, it is still unknown if TDG/BER-dependent demethylation is operational during other types of cellular differentiation and whether it represents a general mechanism of rearrangement of the DNA methylation patterns during specification and commitment of post-mitotic somatic cell types in mammals.

In the present study we aimed to determine the dynamics of enzymatic oxidation of 5mC as well as the expression of transcripts of DNA demethylation-associated proteins during differentiation of human pluripotent stem cells (hPSCs) into hepatic endoderm.

## Results

**Figure 1.**
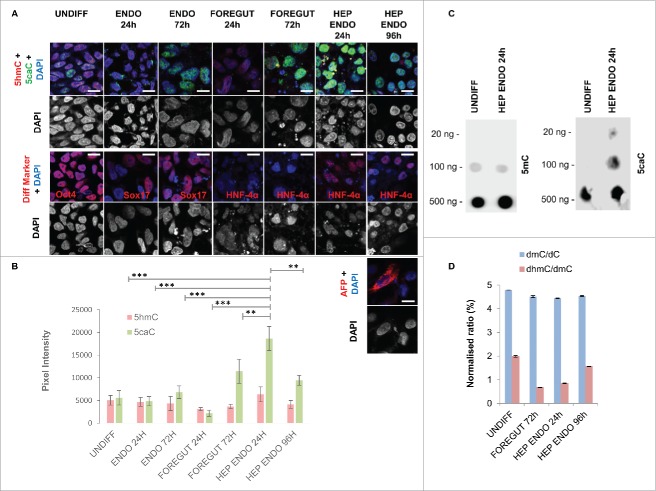
Dynamics of enzymatic 5mC oxidation during hepatic differentiation. (A) Co-detection of 5caC with 5hmC and DAPI (upper row) or of the indicated differentiation markers with DAPI (middle row and lower row for HEP ENDO 96 h stage) in undifferentiated REBL-PAT hiPSCs and at specified stages of their differentiation toward hepatic endoderm. Cell cultures were immunostained in parallel under the same experimental conditions and imaged at identical settings. UNDIFF – undifferentiated cells; ENDO 24 h and 72 h – cells 24 and 72 h after definitive endoderm induction; FOREGUT 24 h and 72 h – cells 24 and 72 h after induction of foregut endoderm; HEP ENDO 24 h and 96 h – cells 24 and 96 h after induction of hepatic endoderm. Merged views and individual channel for DAPI are shown. Scale bars are 15 µm. (B) Quantification of 5 hmC and 5caC signal intensities in REBL-PAT hiPSCs at the specified stages of their differentiation into hepatocytes. Experimental error is shown as SD ***P < 0.001; **P<0.01. (C) DNA dot blot of 5caC and 5mC in undifferentiated hiPSCs and in differentiating cells 24 h after induction of hepatic endoderm. The amounts of DNA loaded on to membranes are indicated. (D) dmC/dC and dhmC/dmC ratios obtained from the quantification of MS peaks in undifferentiated hiPSCs and at indicated stages of their differentiation toward foregut and hepatic endoderm. Experimental error is shown as SD.

To examine the global levels of oxi-mCs during hepatic differentiation, we used a recently published protocol that directs differentiation of hPSCs into a homogenous population of fetal-like hepatocyte cells.^21,22^ This protocol mimics liver embryonic development and comprises 4 stages: differentiation of hPSCs into definitive endoderm (stage 1), differentiation of definitive endoderm cells into anterior definitive or foregut endoderm (stage 2) and differentiation of foregut precursors into hepatic progenitors (stage 3) followed by functional maturation of the obtained population of hepatocyte-like cells (stage 4).^21^ Initially, we performed co-detection of 5hmC with 5caC in undifferentiated hPSCs and differentiating cells 24 and 72 h after induction of definitive endoderm, 24 and 72 h after foregut induction, or 24 and 96 h after induction of hepatic progenitors using a protocol for sensitive immunostaining of modified forms of cytosine we had previously developed and validated by mass spectrometry (Fig. 1A).^20,23^ In agreement with our previously published data,^20^ we could detect non-negligible 5caC staining in undifferentiated hPSCs (Fig. 1A). Moreover, we also observed a slight increase in 5caC signal intensity in cells at the stage of definitive endoderm specification/commitment that corresponded to the activation of Sox17 expression 72 h after induction of endodermal differentiation (Fig. 1A). However, 5caC signal intensity significantly increased during specification of multipotent foregut precursors (72 h after the induction of foregut endoderm) and peaked 24 h after induction of their differentiation into hepatic endoderm concurrently with the appearance of strong staining for hepatocyte nuclear factor 4 α (HNF-4α) expressed in a range of multipotent endodermal progenitors (Fig. 1A). Importantly, the 5caC immunostaining intensity dropped in differentiating hepatocyte-like cells simultaneously with the onset of expression of α fetoprotein (AFP), a marker of committed hepatic progenitors (Fig. 1A).

Next, we compared the intensities of 5hmC and 5caC signals between the cells at different stages of differentiation via quantification of the corresponding signal intensity profiles in multiple cells (Fig. 1B). This approach demonstrated that, whereas the changes in the levels of 5hmC signal were not very pronounced between all the differentiation stages, 5caC signal in differentiating hepatocyte progenitors 24 h after the induction of hepatic endoderm was significantly *(P* < 0.01 to *P* < 0.001) higher than that in other analyzed cell types (Fig. 1B). Next, we confirmed that the cells at 24 h after hepatic endoderm induction display increased levels of 5caC compared with undifferentiated hPSCs in our dot blot experiments (Fig. 1C). Importantly, the changes in 5caC staining we observed were not associated with any global demethylation event. Thus, according to the results of mass spectrometry (MS) detection of DNA modifications, 5mC content did not alter dramatically between undifferentiated cells and foregut or hepatocyte progenitors (Fig. 1D). Contrasting with 5mC, MS-determined 5hmC levels were dynamic with substantial drop in the ΔhmC/ΔmC ratio in foregut precursors (72 h after induction of foregut endoderm) compared with undifferentiated cells followed by gradual accumulation of this modification during specification of hepatic endoderm (Fig. 1D).

**Figure 2.**
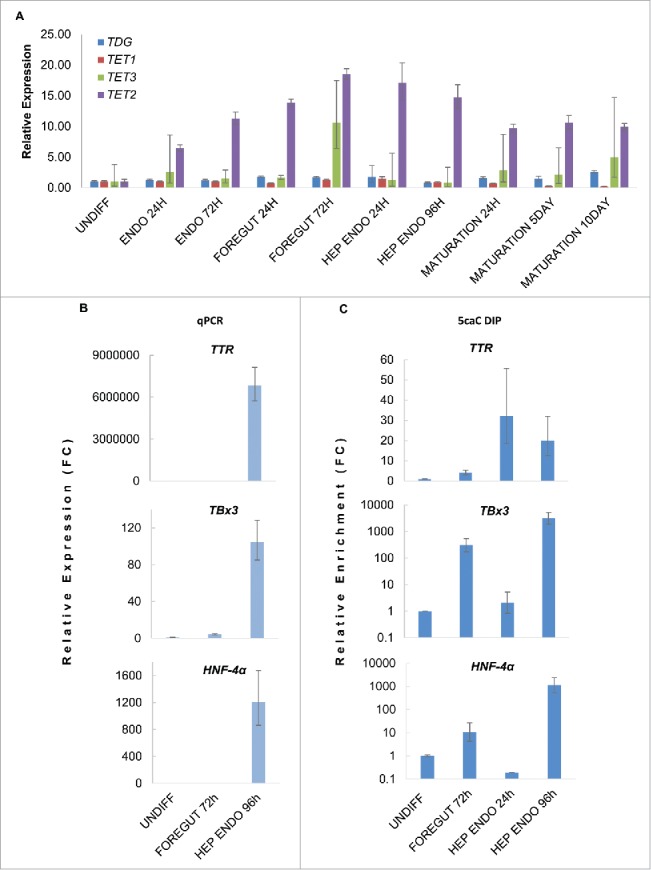
5caC accumulates at promoter regions of hepatocyte markers at the onset of their expression. (A) Relative expression of *TET1/2/3* and *TDG* mRNAs at the specified stages of hepatic differentiation. In addition to the stages of differentiation presented in Fig. 1, 3 stages of hepatocytes maturation (MATURATION) are also shown. (B) Relative expression (Fold change, FC) of the specified hepatocyte markers at the indicated stages of hepatic differentiation. (C) 5caC DIP of indicated promoters in cells at specified stages of hepatic differentiation. *TBx3* and *HNF-4α* DIP results are shown using log scale. Experimental error is presented as SD.

To examine the potential relationship between 5caC accumulation and the components of DNA demethylation machinery, we examined the levels of *TET1/2/3* and *TDG* transcripts in the cells at different stages of their differentiation into hepatic endoderm as well as in hepatocytes that had undergone functional maturation (Fig. 2A). This analysis revealed that *TDG* expression was not substantially changing during the course of the differentiation; however, *TET2* expression peaked at the stages where we witnessed the widespread oxidation of 5mC to 5caC during specification of foregut and commitment of hepatic endoderm suggesting that this protein may be responsible for accumulation of 5caC at these differentiation stages (Fig. 2A).

Since we observed accumulation of 5caC at CpG-rich promoter regions of genes involved in glial and neural specification in differentiating NSCs in our previous study, we decided to check if 5caC is detectable on regulatory regions of genes expressed during hepatic commitment. Given that several mRNAs (*TTR, TBx3, HNF-4α, A1AT, ALB*) were present at relatively high levels during specification of hepatocytes but were essentially absent in multipotent foregut progenitors (Fig. 2B, Fig. S1A), we tested the levels of 5caC at CpG-rich promoter regions of the corresponding genes using 5caC DNA immunoprecipitation (DIP). These experiments revealed that the 5caC levels on *TBx3* and *HNF-4α* promoters dramatically increase 96 h after induction of hepatic endoderm compared with earlier analyzed stages of hepatocyte differentiation and undifferentiated hiPSCs (Fig. 2C). We were also able to detect a less pronounced enrichment of 5caC on the CpG-enriched regions of *TTR* and *A1AT* promoters 24 and 96 h after induction of specification of hepatic endoderm (Fig. 2C, Fig. S1B). As the accumulation of 5caC at the promoter regions of genes expressed during hepatic specification largely corresponded to the onset of their expression in our system, these results together with our previously published NSCs-based data^20^ may imply an involvement of 5mC oxidation to 5caC in transcriptional activation and/or maintenance of transcriptionally active state of lineage-specific genes during differentiation.

**Figure 3.**
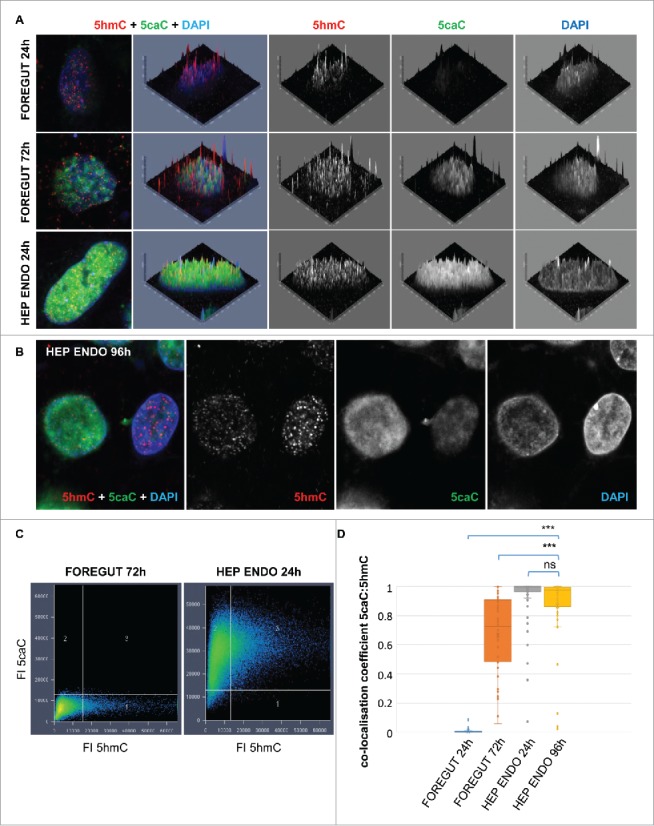
Nuclear distribution of 5hmC and 5caC during specification of foregut endoderm and during hepatic endoderm commitment. (A) Distribution of 5caC, 5hmC, and DAPI signals in the nuclei of representative cells at indicated stages of differentiation after induction of foregut (FOREGUT 24 h, FOREGUT 72 h) and hepatic endoderm (HEP ENDO 24 h). Merged views together with corresponding 2.5XD signal intensity plots and 2.5XD signal intensity plots for individual channels are presented. (B) 5hmC, 5caC and DAPI signals in 2 cells exhibiting 5caC staining of different intensities 96 h after induction of hepatic endoderm. Merged view and individual channels are shown. (C) 5caC/5hmC FI (fluorescence intensity) colocalization plots for representative images of the nuclei of FOREGUT 72 h and HEP ENDO 24 h cells depicted in (A). (D) Boxplot showing 5caC:5hmC colocalization coefficient values for cell populations at the indicated differentiation stages. Twenty to sixty individual cells were analyzed for each stage. ***P < 0.0001, ns – not statistically significant.

We previously found that 5hmC and 5caC were distributed in a semi-overlapping manner in the majority of cells of the murine embryonic brain at 13.5 d post coitum (dpc) stage, which implied that specific genomic regions are subjected to oxidation of 5mC to 5caC during NSCs specification.^20^ Thus, we attempted to assess the nuclear distribution of 5hmC and 5caC in the cells differentiating toward hepatocyte progenitors at the stages of differentiation we observed the accumulation of 5caC (Fig. 3, A-C). Analysis of our confocal images revealed that, similar to the cells of mouse embryonic brain at 13.5 dpc, 5hmC and 5caC were distributed in a semi-overlapping manner during specification/commitment of foregut multipotent progenitors (72 h after induction of foregut endoderm), but these marks displayed very high degrees of spatial overlap 24 h after induction of differentiation of hepatic endoderm (Fig. 3A, C). Correspondingly, the analysis of colocalization of 5caC and 5hmC signals in multiple cells showed that 5caC:5hmC colocalization coefficient values for differentiating cells after induction of hepatic endoderm were significantly higher than those of the multipotent foregut progenitors (Fig. 3D). Such distribution of 5hmC and 5caC signals infers the genome-wide character of 5mC/5hmC oxidation to 5caC in the cells having undergone hepatic specification suggesting that this process is surgical on a wide range of genomic sequences likely including different classes of repetitive DNA that comprise approximately half of the human genome. Moreover, in the culture of hepatocyte progenitors 96 h after induction of hepatic differentiation, we found cells with 2 types of 5caC nuclear distribution: cells displaying high levels of this mark immunostaining accompanied by a relatively low 5hmC signal and cells with virtually undetectable 5caC and comparatively high 5hmC staining intensity (Fig. 3B). We concluded that these staining results are likely to reflect the onset of the general decrease in 5caC content occurring at this stage with different cells loosing this mark at slightly different time points due to asynchrony of the differentiating cell cultures.

**Figure 4.**
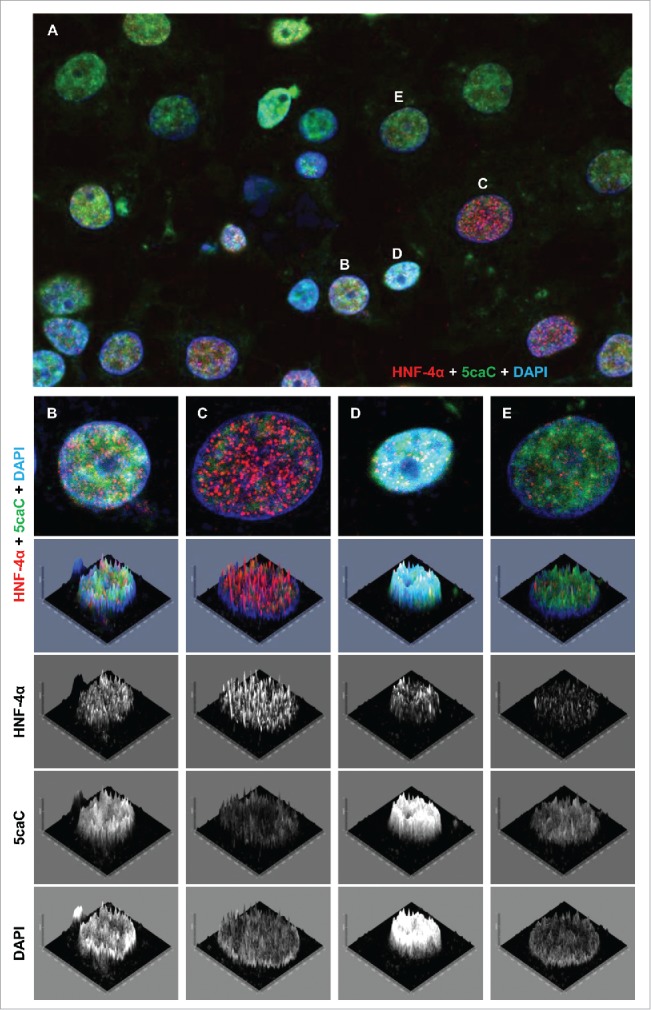
5caC staining intensity does not correlate with the levels of HNF-4α expression during hepatic endoderm commitment. (A) Co-detection of 5caC with HNF-4α and DAPI in the culture of differentiating cells 96 h after induction of hepatic endoderm. Merged view is shown. Individual nuclei with different levels of 5caC and HNF-4α staining presented in (B-E) are marked. (B-E) Distribution of 5caC, HNF-4α, and DAPI staining in 4 individual nuclei exhibiting different intensities of 5caC and HNF-4α signals. Merged views together with corresponding 2.5XD signal intensity plots and 2.5XD signal intensity plots for individual channels are presented.

**Figure 5.**
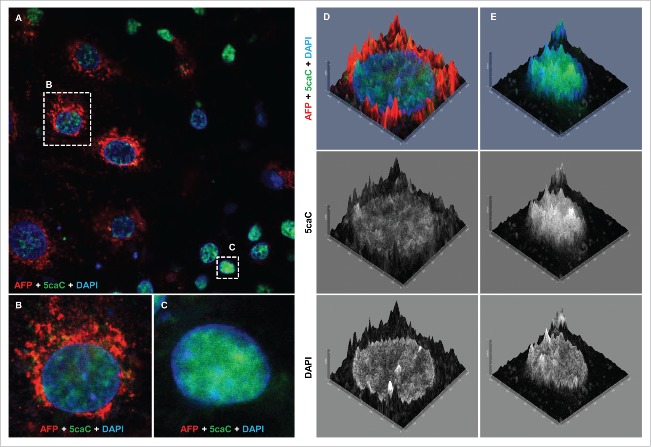
The levels of 5caC staining drop concurrently with the onset of AFP expression during hepatic endoderm commitment. (A) Co-detection of 5caC with AFP and DAPI in the culture of differentiating cells 96 h after induction of hepatic endoderm. Merged view is shown. Individual cells with different levels of 5caC and AFP staining presented in (B) and (C) are marked with dotted rectangles. (B-C) Co-detection of 5caC with AFP and DAPI in individual AFP-positive (B) and negative (C) cells. Merged views are shown. (D-E) 2.5XD signal intensity profiles generated for AFP-positive (D) and negative (E) cells shown in (B-C). Merged views alongside individual channels for 5caC and DAPI are shown.

To test if this heterogeneity of 5caC staining corresponded to the expression of any specific markers of differentiation in these cells, we performed co-immunostaining of 5caC with HNF-4α and AFP in hepatocyte progenitors 96 h after induction of the hepatic endoderm. Importantly, we could not find any correlation between the levels of 5caC immunostaining and the intensity of HNF-4α signal in these cells (Fig. 4). Thus, the culture of differentiating hepatic progenitors contained cells with high 5caC signal accompanied by high levels of HNF-4α staining (Fig. 4B), cells with high levels of HNF-4α expression and virtually undetectable 5caC (Fig. 4C), cells with increased 5caC staining and relatively low HNF-4α expression (Fig. 4D) together with cells where both signals were fairly moderate (Fig. 4E). In contrast with these immunostaining experiments, the intensity of 5caC staining negatively correlated with expression of AFP in differentiating hepatic progenitors (Fig. 5). Thus, the 5caC signal was low or undetectable in AFP-positive cells (Fig. 5B, D), whereas AFP-negative hepatocyte progenitors were characterized by pronounced 5caC immunostaining (Fig. 5C, E). Given that *HNF-4α* is expressed in a wide range of multi- and uni-potent precursors of endodermal lineages and AFP expression is a characteristic of committed hepatic progenitors, our results imply that the global 5caC levels start to decline simultaneously with the onset of expression of markers of hepatocytes commitment. Thus, we concluded that the transient accumulation of 5caC we observe is likely linked with reorganization of the patterns of DNA methylation occurring during final stages of specification of hepatic lineage.

## Discussion

DNA methylation patterns are rearranged during several key stages of the mammalian lifecycle. Specifically, a wave of genome-wide DNA demethylation occurs in pre-implantation embryos, DNA methylation is globally erased during maturation of PGCs, and the 5mC profiles are reorganized during differentiation of somatic cell types.^1,2^ Whereas the involvement of TDG/BER-dependent demethylation in resetting the zygotic and germ cell DNA methylation patterns is currently perceived as unlikely or, at best, questionable, numerous lines of experimental evidence suggest the importance of both TET proteins and TDG for, at least, some types of cellular differentiation.^13^ Thus, combined depletion of all 3 TET proteins compromises proper differentiation of mouse ESCs.^24^ Moreover, TET-dependent 5mC oxidation has been implicated in the modulation of enhancer activity during differentiation.^25,26^ Importantly, TDG is required for proper neuronal differentiation *in vitro*^27^ and, according to a recent report, its knockdown affects differentiation of pig preadipocytes.^28^ Furthermore, TDG knockout embryos die at 11.5 dpc, the developmental stage of active organogenesis when differentiation of various types of cellular progenitors is underway.^27,29^ In line with this, our previous study demonstrated transient accumulation of 5fC and 5caC during lineage specification of NSCs, at initial stages of their differentiation toward neuronal and glial lineages, and prospective involvement of TDG in removal of these marks from DNA during this process.^20^ In this context, our finding that hepatocyte progenitors experience a wave of 5caC accumulation during their specification and in advance of the onset of expression of such markers of committed hepatic progenitors as AFP, may suggest that TDG/BER-dependent active DNA demethylation governs rearrangement of the DNA methylation patterns at the transition from a progenitor to an early functional hepatocyte state. Moreover, since we observe the transitory increase in 5caC levels in 2 such different types of differentiation as neural/glial and endodermal/hepatic, the active DNA demethylation by DNA repair may represent a general mechanism used for reorganization of the 5mC profiles during terminal differentiation of somatic cell types in mammals.

Although the involvement of TDG/BER-dependent demethylation in hepatocyte differentiation is highly likely, it is important to note, in our opinion, that the functional significance of TDG for elimination of 5caC in this system is yet to be tested. Moreover, unlike embryonic brain development,^20^ the levels of *TDG* transcript do not follow the dynamics of 5caC throughout hepatic differentiation, according to our results. Therefore, we cannot exclude a possibility that a TDG-independent mechanism of 5caC removal from DNA is operational in differentiating hepatocyte progenitors. From this perspective, it is interesting that, whereas the knockouts of DNA glycosylases other than TDG do not seem to interfere with the developmental capacity,^30^ an unidentified DNA decarboxylase activity, potentially capable of converting 5caC to unmodified cytosine, is detectable in mouse ESCs by isotope tracing.^31^

Despite both 5fC and 5caC may potentially serve as intermediates in active TDG-dependent DNA demethylation, several recent reports suggest that all the oxi-mCs may also act as proper epigenetic marks playing their own specific roles in transcriptional regulation.^32,33,34^ Thus, both 5fC and 5caC are associated with specific sets of regulatory sequences in the genome^20,35^ and potentially interact with distinct groups of candidate “reader” proteins identified for each of the oxi-mCs in mass spectrometry-based experiments.^36^ Moreover, such potential “reader” proteins for 5fC and 5caC comprise chromatin remodeling proteins, transcription factors, and histone modifying enzymes.^36^ Therefore, our results may not only suggest that active demethylation controls the rearrangement of 5mC profiles during specification/commitment of hepatic progenitors but may also point to a possibility that the transient presence of 5caC in specific regulatory genomic regions affects transcriptional activity of the corresponding genes in differentiating cells via 5caC-dependent recruitment of transcriptional factors or chromatin modifying complexes, contributing to the differentiation stage-specific patterns of gene expression.

Summarizing, our data imply involvement of TDG/BER-dependent demethylation and/or 5caC-dependent regulation of transcription in specification of foregut endoderm and commitment of hepatocytes. We show that transient 5caC accumulation is a common feature of both neural/glial and endoderm/hepatic differentiation. This suggests that oxidation of 5mC may represent a general mechanism of rearrangement of 5mC profiles used during lineage specification of post-mitotic cells in mammals.

## Materials and methods

### hiPSCs culture and differentiation

REBL-PAT hiPSCs (R-Pat) hiPSCs were maintained in Essential 8™ (E8) medium with supplement (#A1517001) on Matrigel™-coated (34.7 µg/cm^2^) T25 tissue culture flasks at 37°C with 5% CO2. Cells were passaged every 3–4 d using TrypLE™ Select Enzyme (#12563029). R-Pat hiPSCs were reprogrammed from skin fibroblasts using Sendai virus by Gary Duncan at the University of Nottingham. Differentiation of hiPSCs to hepatocyte-like cells was performed according to previously published protocol.^21^

### Immunocytochemistry, confocal microscopy and image quantification

Immunochemistry was performed as described previously.^20^ The samples were incubated in 2 N HCl for 1 h at 37°C. Anti-5hmC mouse monoclonal (Active Motif, 1:5000 dilution), anti-5caC rabbit polyclonal (Active Motif, 1:500 dilution), anti-HNF-4α mouse monoclonal (Santa Cruz Biotechnology), anti-Oct4 mouse monoclonal (Santa Cruz Biotechnology), anti-AFP mouse monoclonal (Abcam, #ab3969) and anti-Sox17 goat polyclonal (R&D Systems AF1924-SP) primary antibodies were used for immunochemistry. Peroxidase-conjugated anti-rabbit secondary antibody (Dako) and the tyramide signal enhancement system (Perkin Elmer, 1:200 dilution, 3 min of incubation with tyramide) were used for 5caC detection. 5hmC and protein differentiation markers were visualized using 555-conjugated secondary antibody (Alexafluor). Control staining without primary antibody produced no detectable signal. Images (500 nm optical sections) were acquired with a Zeiss LSM 700 AxioObserver confocal microscope using a Plan-Apochromat 63x/1.40 Oil DIC M27 objective and processed using Image J and Adobe Photoshop. 2.5XD signal intensity plots and profiles were generated using ZEN Zeiss LSM 700 imaging software. Colocalization coefficients were determined using the inbuilt analysis function of ZEN with a threshold intensity of 50. The significance was determined by one way ANOVA and post hoc Dunnett test, ***P < 0.0001. Confocal raw data are available upon request. For quantification of the 5hmC and 5caC signal intensities, mean values of the average of 4 intensity profiles generated across 10–12 nuclei were calculated for each differentiation stage. Statistical significance was determined using 2-tailed t-test following assessment of the variance with F-test.

### Mass spectrometry

DNA samples were digested to nucleosides based on a reported method.^37^ LC-MS/MS analysis was performed in duplicate by injecting digested DNAs onto an Agilent 1290 UHPLC equipped with a G4212A diode array detector and a 6490A Triple Quadrupole Mass Detector operating under positive electrospray ionization mode (+ESI). UHPLC was performed using a Waters XSelect HSS T3 XP column (2.1 × 100 mm, 2.5 μm) with a gradient mobile phase consisting of aqueous ammonium formate (10 mM, pH 4.4) and methanol. MS data acquisition was performed in the dynamic multiple reaction monitoring (DMRM) mode. Each nucleoside was identified and quantified in the extracted chromatogram associated with its specific MS/MS transition: dC [M+H]+ at m/z 228 →112, dmC [M+H]+ at m/z 242→126, and dhmC [M+H]+ at m/z 258→142. External calibration curves with known amounts of the nucleosides were used to calculate their ratios within the samples analyzed.

### Dot blots

Dot blots were performed as described previously^20^ using 5caC rabbit polyclonal (Active Motif, 1:1000 dilution) and 5mC rabbit polyclonal (Cell Signaling Technology, D3S2Z, #28692, 1:5000 dilution) antibodies. Equal dilutions of DNA were loaded onto membranes.

### 5caC-DNA IP (DIP)

Genomic DNA was isolated according to standard procedures and sonicated using Diagenode Bioruptor Standard UCD-200. Genomic DNA (2 μg) was used for immunoprecipitation. 5caC-DIP was performed as described^20^ using rabbit polyclonal 5caC (Active Motif) antibody and magnetic anti-rabbit Dynabeads (Invitrogen). Samples were purified using Qiagen DNA purification kit and analyzed by real-time PCR (RT-PCR) performed with SYBR Green PCR Master Mix (GoTaq, Promega) according to standard procedures. Fold enrichment was calculated as 2^−ΔΔCt^, where ΔCt = Ct(enriched)-Ct(input) and ΔΔCt = ΔCt – Ct (no antibody). Experimental error is expressed as SD. The following primers were used for RT-PCR: *TTR*: CATGAACAAAGCCACGCATG and ATTCTTTTCCTCCTGGCCGA; *TBx3*: TGAGGCATTTCAGACGTGGG and ATCGGTACTACTGCCTGTCC; *HNF-4α*: TGGTAGAGACGGGGTTTCAC and ACCTTCAGCCCCTACAGATG; *A1AT*: AAGGGAGAGGGTGACTTGTC and AAGTAGACTTCGGGTGGAGG; *ALB*: TGGCTCATGACTGTAATCCCA and AGTTCACGCCATTCTTCTGC.

### Gene expression analysis

Expression of *TET1/2/3, TDG* and hepatocyte markers was analyzed by quantitative PCR, according to standard procedures. Gene expression was normalized by comparison to levels of *GAPDH* gene expression. The following primers were used:

*TET1*: CTTGGTATGAGTGGGAGTG and GAGCATTAAAGGTAGCAATTG;
*TET2*: GCAAGATCTTCTTCACAG and GCATGGTTATGTATCAAGTA;
*TET3*: CTCTGAAGTCAGAGGAGAA and GTCCAGGAAGTTGTGTTC;
*TDG*: CAGCTATTCCCTTCAGCA and GGAACTTCTTCTGGCATTTG;
*GAPDH*: GATGCTGGCGCTGAGTACG and GCAGAGATGATGACCCTTTTGG

Primers used for analysis of expression of the hepatocyte markers are available upon request.

## Supplementary Material

KEPI_A_1292189_s02.pdf
