# Supplementary material for: Dynamics of 5-carboxylcytosine during hepatic differentiation: Potential general role for active demethylation by DNA repair in lineage specification
Source: Epigenetics. 2017 Mar 7;12(4):277–86. doi: 10.1080/15592294.2017.1292189 (PMC5398770; doi:10.1080/15592294.2017.1292189)
Supplement: KEPI_A_1292189_s02.pdf [file kepi-12-04-1292189-s001.pdf]

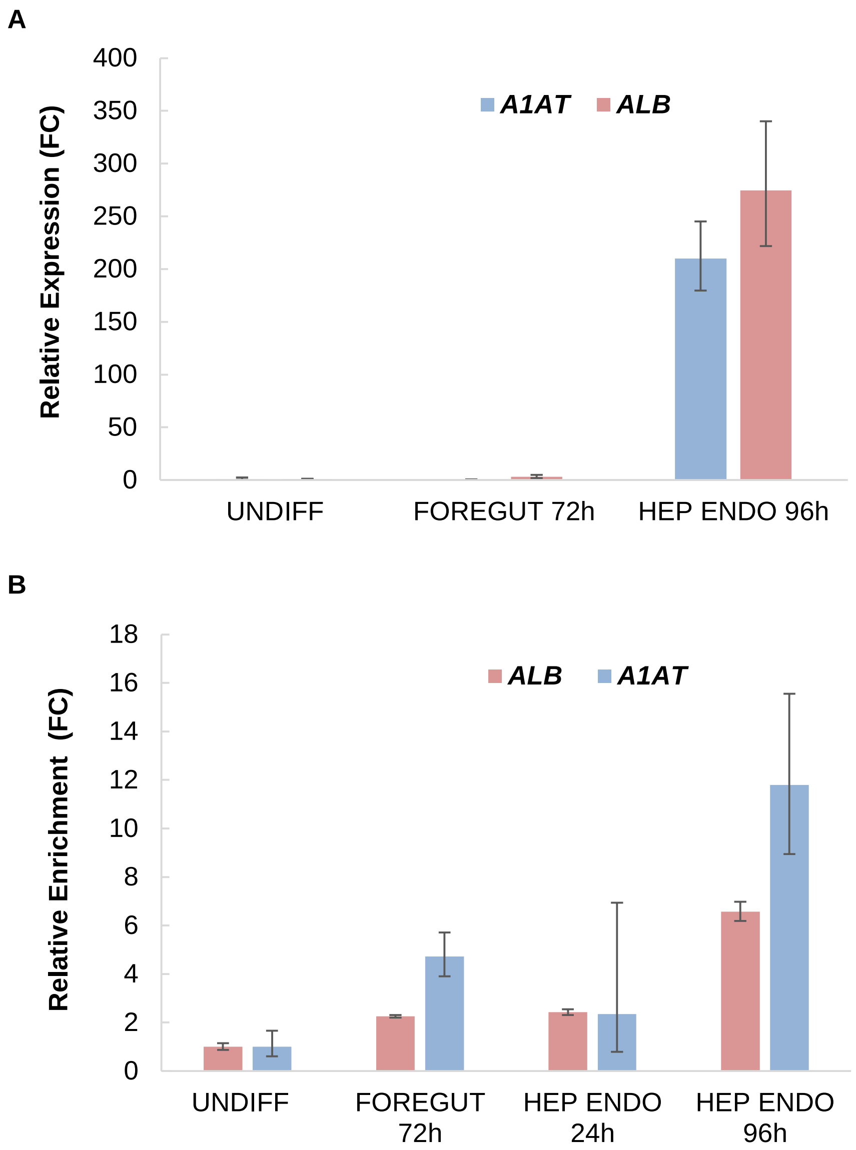

Supplementary Figure S1. (A) Relative expression of the specified hepatocyte markers at the indicated stages of hepatic differentiation. (B) 5caC DIP of indicated promoters in the cells at specified differentiation stages. Experimental error is presented as S. D.
